# Supplementary material for: Tafenoquine following G6PD screening versus primaquine for the treatment of vivax malaria in Brazil: A cost-effectiveness analysis using a transmission model
Source: PLoS Med. 2024 Jan 9;21(1):e1004255. doi: 10.1371/journal.pmed.1004255 (PMC10775976; doi:10.1371/journal.pmed.1004255)
Supplement: S1 CHEERS Checklist — (DOCX) [file pmed.1004255.s001.docx]

# CHEERS 2022 Checklist

| **Topic** | **No.** | **Item** | **Location where item is reported** |
| --- | --- | --- | --- |
| **Title** |  |  |  |
|  | 1 | Identify the study as an economic evaluation and specify the interventions being compared. | a cost-effectiveness analysis using a transmission model |
| **Abstract** |  |  |  |
|  | 2 | Provide a structured summary that highlights context, key methods, results, and alternative analyses. | Background: Malaria transmission modelling has demonstrated the potential impact of semi-quantitative glucose-6-phosphate dehydrogenase (G6PD) testing and treatment with single-dose tafenoquine for Plasmodium vivax radical cure but has not investigated the associated costs. This study evaluated the cost-effectiveness of P. vivax treatment with tafenoquine after G6PD testing using a transmission model. Methods and Findings: We explored the cost-effectiveness of using tafenoquine after G6PD screening as compared to usual practice (7-day low-dose primaquine (0.5 mg/kg/day) without G6PD screening) in Brazil using a 10-year time horizon with 5% discounting considering four scenarios: 1) tafenoquine for adults only assuming 66.7% primaquine treatment adherence, 2) tafenoquine for adults and children aged >2 years assuming 66.7% primaquine adherence, 3) tafenoquine for adults only assuming 90% primaquine adherence, 4) tafenoquine for adults only assuming 30% primaquine adherence. The incremental cost-effectiveness ratios (ICERs) were estimated by dividing the incremental costs by the disability-adjusted life-years (DALYs) averted. These were compared to a willingness to pay threshold of US$7,800 for Brazil, and one-way and probabilistic sensitivity analyses were performed. All four scenarios were cost-effective in the base case analysis using this willingness to pay threshold with ICERs ranging from US$154–1836. One-way sensitivity analyses showed that the results were most sensitive to severity and mortality due to vivax malaria, the lifetime and number of semi-quantitative G6PD analysers needed, cost per malaria episode and per G6PD test strips, and life expectancy. All scenarios had a 100% likelihood of being cost-effective at the willingness to pay threshold. The main limitations of this study are due to parameter uncertainty around our cost estimates for low transmission settings, the costs of G6PD screening, and the severity of vivax malaria Conclusions: In our modelling study that incorporated impact on transmission, tafenoquine prescribed after a semi-quantitative G6PD testing was highly likely to be cost-effective in Brazil. These results demonstrate the potential health and economic importance of ensuring safe and effective radical cure. |
| **Introduction** |  |  |  |
| **Background and objectives** | 3 | Give the context for the study, the study question, and its practical relevance for decision making in policy or practice. | The impact of tafenoquine following G6PD screening on transmission in Brazil has been explored in a recent mathematical model [19]. The model simulations demonstrated a decrease in transmission over a ten-year time horizon, although this was not sufficient to achieve elimination. Since a paediatric formulation of tafenoquine is being developed, a scenario is included to evaluate the impact of prescribing to adults and children over the age of six months in addition to an adults only scenario. Here, we conduct a complementary economic evaluation with the transmission model predictions to explore the impact on the overall cost-effectiveness of a semi-quantitative G6PD test-and-treat strategy using tafenoquine for radical cure. |
| **Methods** |  |  |  |
| **Health economic analysis plan** | 4 | Indicate whether a health economic analysis plan was developed and where available. | A health economic analysis plan was not developed for this model-based analysis. |
| **Study population** | 5 | Describe characteristics of the study population (such as age range, demographics, socioeconomic, or clinical characteristics). | The cost-effectiveness analysis presented herein uses the median value of multiple disease and treatment model states across 100 simulations of the individual-based model over time for each municipality. Specifically, the number of primaquine and tafenoquine doses, cases in males and females over 16 years, cases in males and females under 16 years, number of cases in pregnant women, and G6PD tests were provided by the transmission model. The model assumed a truncated exponential age distribution with a mean age of 32 years. Under this model 15.6% of those under 16 years would be under the age of 2 years and therefore be ineligible for tafenoquine. Approximately 4% of those under 16 years would be under the age of 6 months and not receive radical cure, while the remainder of children under 2 years would receive primaquine. |
| **Setting and location** | 6 | Provide relevant contextual information that may influence findings. | The model presented in White et al. was extended in Nekkab et al. to the Brazilian context, to capture the two predominant modes of transmission: peri-domestic transmission within and near households, and; occupational exposure predominantly affecting working age males. The model was extended to capture these modes of transmission with: the stratification of individuals in the model as male or female; males further stratified according to their exposure source (peri-domestic or occupational), and; two populations of mosquitoes representing peri-domestic or occupational exposure. The model was calibrated to the Brazilian context using several data sources from Brazil and validated by the National Malaria Control Programme and malaria experts in Brazil. The model was calibrated to case notification data for 126 municipalities for which there were at least 100 cases in 2018, such that heterogeneity in demographics and transmission intensity was represented. The intervention simulations were generated for a population size of 100,000 and subsequently converted to the appropriate population size for each municipality estimated for 2018. |
| **Comparators** | 7 | Describe the interventions or strategies being compared and why chosen. | The original transmission model analysis explored six treatment policy scenarios for tafenoquine following G6PD screening [19]; each of which were compared to the baseline scenario of seven-day low-dose primaquine (3.5mg/kg total) for all eligible patients without G6PD screening. Here we explore the cost-effectiveness of four of those scenarios. Scenario 1 explored tafenoquine for adults over the age of 16 (tafenoquine for adults), while Scenario 2 expanded access to children who were over the age of 2 years (tafenoquine for all). While Scenarios 1 and 2 assumed that adherence to the seven-day primaquine regimen was 66.7% [8], Scenarios 3 (high primaquine adherence) and 4 (low primaquine adherence) explored the impact of assuming 90% and 30% pre-existing primaquine adherence when prescribing tafenoquine to adults, respectively. |
| **Perspective** | 8 | State the perspective(s) adopted by the study and why chosen. | Costs from a healthcare provider perspective are reported |
| **Time horizon** | 9 | State the time horizon for the study and why appropriate. | The results cover a ten-year time horizon from 2020 to 2029 |
| **Discount rate** | 10 | Report the discount rate(s) and reason chosen. | Both costs and outcomes were discounted at 5% per year in the base case analysis to reflect the value that society attaches to present consumption as opposed to consumption in the future in Brazil. |
| **Selection of outcomes** | 11 | Describe what outcomes were used as the measure(s) of benefit(s) and harm(s). | DALYs for each scenario were calculated by adding the years of life lost to the years of life with disability |
| **Measurement of outcomes** | 12 | Describe how outcomes used to capture benefit(s) and harm(s) were measured. | Model parameters for years of life with disability are shown in Table 1. It was assumed that all malaria cases resulted in anaemia. The average age of patients with vivax malaria by sex were derived from the SIVEP database [23]. These ages matched to the life expectancy for that age and sex to calculate the years of life lost [28]. Life expectancy for both sexes was varied by ±10% in the sensitivity analyses. |
| **Valuation of outcomes** | 13 | Describe the population and methods used to measure and value outcomes. | N/A |
| **Measurement and valuation of resources and costs** | 14 | Describe how costs were valued. | Table 1 provides the unit costs and further information about how these costs were applied. The cost of radical cure was also included for severe cases, but it was assumed this would be prescribed after release from the hospital. The semi-quantitative machine was assumed to have a lifetime of five years (range: 3 to 10). The number of healthcare facilities in each municipality was calculated from the 2018 Malaria Epidemiological Surveillance Information System (SIVEP) data on number of health units that saw at least 100 cases of vivax malaria [2, 23]. In the base case, it was assumed that on average 1.05 machines (range: 1 to 2) would be needed per healthcare facility. |
| **Currency, price date, and conversion** | 15 | Report the dates of the estimated resource quantities and unit costs, plus the currency and year of conversion. | When applicable, costs were inflated to 2020 using gross domestic product deflators [26] and converted from Brazilian reais to US$ [27]. |
| **Rationale and description of model** | 16 | If modelling is used, describe in detail and why used. Report if the model is publicly available and where it can be accessed. | The cost-effectiveness analysis presented herein uses the median value of multiple disease and treatment model states across 100 simulations of the individual-based model over time for each municipality. Specifically, the number of primaquine and tafenoquine doses, cases in males and females over 16 years, cases in males and females under 16 years, number of cases in pregnant women, and G6PD tests were provided by the transmission model. The model assumed a truncated exponential age distribution with a mean age of 32 years. Under this model 15.6% of those under 16 years would be under the age of 2 years and therefore be ineligible for tafenoquine. Approximately 4% of those under 16 years would be under the age of 6 months and not receive radical cure, while the remainder of children under 2 years would receive primaquine....The model code is available in Appendix 1. |
| **Analytics and assumptions** | 17 | Describe any methods for analysing or statistically transforming data, any extrapolation methods, and approaches for validating any model used. | The intervention simulations were generated for a population size of 100,000 and subsequently converted to the appropriate population size for each municipality estimated for 2018. The results cover a ten-year time horizon from 2020 to 2029, and assume that G6PD screening is fully rolled out in January 2021. |
| **Characterising heterogeneity** | 18 | Describe any methods used for estimating how the results of the study vary for subgroups. | N/A |
| **Characterising distributional effects** | 19 | Describe how impacts are distributed across different individuals or adjustments made to reflect priority populations. | N/A |
| **Characterising uncertainty** | 20 | Describe methods to characterise any sources of uncertainty in the analysis. | A one-way sensitivity analysis and probabilistic sensitivity analysis (PSA) was run on all parameters in the cost-effectiveness analyses. Table 1 contains a list of all parameters, with the point estimate used as base case, and lower and upper values used directly in the one-way sensitivity analysis. For the PSA, the point estimate, lower and upper values were used to fit appropriate distributions from which to sample (i.e., by matching the lower and upper values to 2.5- and 97.5-percentiles, respectively). Gamma distributions were used for costs and DALY weights, and beta distributions were used for all other parameters except for the lifetime of the semi-quantitative machine. For this parameter we assumed a normal distribution truncated below at 1 year, on the assumption that the average semi-quantitative device lifetime was at least 1 year. A range of two to ten years was used for the lifetime of the semi-quantitative machine in the one-way sensitivity analysis. Table 1 also contains the two fitted distributional model parameters for each cost-effectiveness analysis model parameter. A total of 10,000 model parameters were sampled from the specified distributions. The PSA model parameters were each applied to the transmission model output for each municipality for the four scenarios, enabling the overall mean costs, DALYs, and ICERs to be calculated and the 2.5 and 97.5 percentiles (referred to herein as the 95% Credible Interval [95%CrI]). |
| **Approach to engagement with patients and others affected by the study** | 21 | Describe any approaches to engage patients or service recipients, the general public, communities, or stakeholders (such as clinicians or payers) in the design of the study. | N/A |
| **Results** |  |  |  |
| **Study parameters** | 22 | Report all analytic inputs (such as values, ranges, references) including uncertainty or distributional assumptions. | See Table 1 |
| **Summary of main results** | 23 | Report the mean values for the main categories of costs and outcomes of interest and summarise them in the most appropriate overall measure. | See Table 2 |
| **Effect of uncertainty** | 24 | Describe how uncertainty about analytic judgments, inputs, or projections affect findings. Report the effect of choice of discount rate and time horizon, if applicable. | See Table 2 |
| **Effect of engagement with patients and others affected by the study** | 25 | Report on any difference patient/service recipient, general public, community, or stakeholder involvement made to the approach or findings of the study | N/A |
| **Discussion** |  |  |  |
| **Study findings, limitations, generalisability, and current knowledge** | 26 | Report key findings, limitations, ethical or equity considerations not captured, and how these could affect patients, policy, or practice. | A limitation of our study is regarding the uncertainty of our cost estimates for low transmission settings due to higher stochasticity of the transmission model and model assumptions. For very low transmission settings, stochastic noise and fadeout result in more unstable transmission dynamics and greater variation between simulations. In addition, calibrated incidence per 1000 population for these low transmission settings assumes homogenous mixing in the population and no importations to sustain transmission; therefore, local transmission dynamics in communities are likely to differ compared to the simplified model aggregated municipality-level assumptions. Consequently, projected DALYs for these settings are less reliable and should be interpreted with caution. The absence of this variation in model output has minimal impact on municipalities with higher transmission (and thus at a national level) where the results converge to those from a deterministic model, and where the majority of the costs and benefits would be accrued. By estimating costs for a large range of transmission settings across Brazil and providing detailed results for three archetype settings with stable transmission, the drivers of impact on costs we identified overall are less impacted by model uncertainty. Appropriately incorporating the costs of semi-quantitative G6PD screening is challenging for a number of reasons. First, the costs of the machine, test strips, and controls have not been confirmed for procurement by the Ministry of Health in Brazil. While we have indicative costs, these may change due to distribution costs, customs, taxes, and any price changes that may occur when negotiating purchase of enough machines to implement nationally in Brazil. Second, the model assumed that G6PD screening can begin everywhere at the same time. It is likely that the roll out will occur gradually and that uptake may be slow or patchy. In addition, it is assumed that a semi-quantitative machine will be placed at health units that had a case of vivax malaria in 2018 throughout the entire time horizon of the analysis. For large units and units that make home visits, more than one machine might be needed; this would increase the costs. Alternatively, health units that do not continue to see malaria cases may not need to continue stocking a semi-quantitative machine. Finally, since transmission is an important driver of results, these findings are dependent on future trends in vivax malaria cases. Severity and mortality of vivax malaria are also challenging parameters to estimate as data are sparse. The base case value of 0.03% used here was from a study of vivax malaria patients admitted to a hospital in Brazil during 2009–11 [13] and from a review that accessed 2014 data on microscopically confirmed malaria cases and related deaths from the National Malaria Prevention and Control Programme, Ministry of Health of Brazil [32]. The latter included falciparum malaria, indicating that it may be an overestimate. Another study of vivax malaria from a tertiary care centre in Manaus from 1996 to 2010 found a lower case fatality rate of 0.01% [15]. To be conservative, our low value for the sensitivity analyses assumed no mortality due to vivax malaria. While this parameter had a large impact on the results in the one-way sensitivity analysis, all scenarios remained cost-effective across all three municipalities when this assumption was applied. Finally, this only compares tafenoquine with low-dose primaquine treatment. While low-dose primaquine (3.5 mg/kg total) is the current recommended treatment in Brazil, a recent comparison of low-dose with a high-dose primaquine regimen (7.0 mg/kg total) found a 27% difference in the percentage of patients who were recurrence-free at day 168 when these regimens were supervised [33]. While tafenoquine has been shown to have similar efficacy to low-dose primaquine [10], it has not been directly compared to high-dose primaquine in Brazil. This clinical comparison would need to be done before the implications for the cost-effectiveness analysis could be ascertained. |
| **Other relevant information** |  |  |  |
| **Source of funding** | 27 | Describe how the study was funded and any role of the funder in the identification, design, conduct, and reporting of the analysis | This work was supported by Medicines for Malaria Ventures. WMM is funded by FAPEAM through POSGRAD and Pró-Estado public calls. WMM and ML are fellows of the National Council for Scientific and Technological Development (CNPq). MTW is funded by The Bill & Melinda Gates Foundation [grant number INV-024368]. The sponsor had no role in the study design, analysis, decision to publish, or preparation of the manuscript. |
| **Conflicts of interest** | 28 | Report authors conflicts of interest according to journal or International Committee of Medical Journal Editors requirements. | The authors have declared that no competing interests exist. |

*From:* Husereau D, Drummond M, Augustovski F, et al. Consolidated Health Economic Evaluation Reporting Standards 2022 (CHEERS 2022) Explanation and Elaboration: A Report of the ISPOR CHEERS II Good Practices Task Force. Value Health 2022;25. <doi:10.1016/j.jval.2021.10.008>
