# Supplementary figures and images for: Tafenoquine following G6PD screening versus primaquine for the treatment of vivax malaria in Brazil: A cost-effectiveness analysis using a transmission model
Source: PLoS Med. 2024 Jan 9;21(1):e1004255. doi: 10.1371/journal.pmed.1004255 (PMC10775976; doi:10.1371/journal.pmed.1004255)

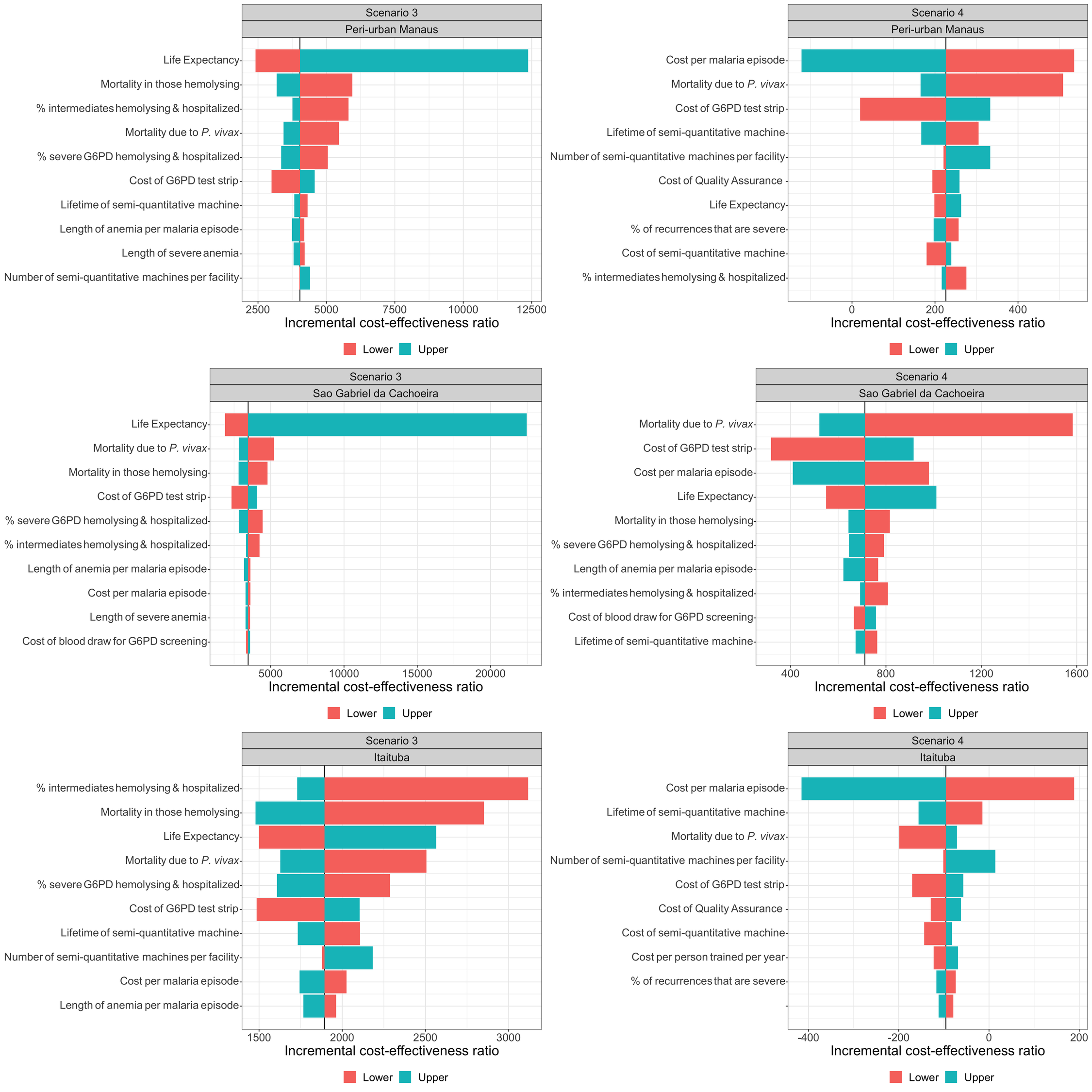

Supplement: S2 Appendix — See Table 1 for the ranges used for this analysis. (TIF) [file pmed.1004255.s003.tif]

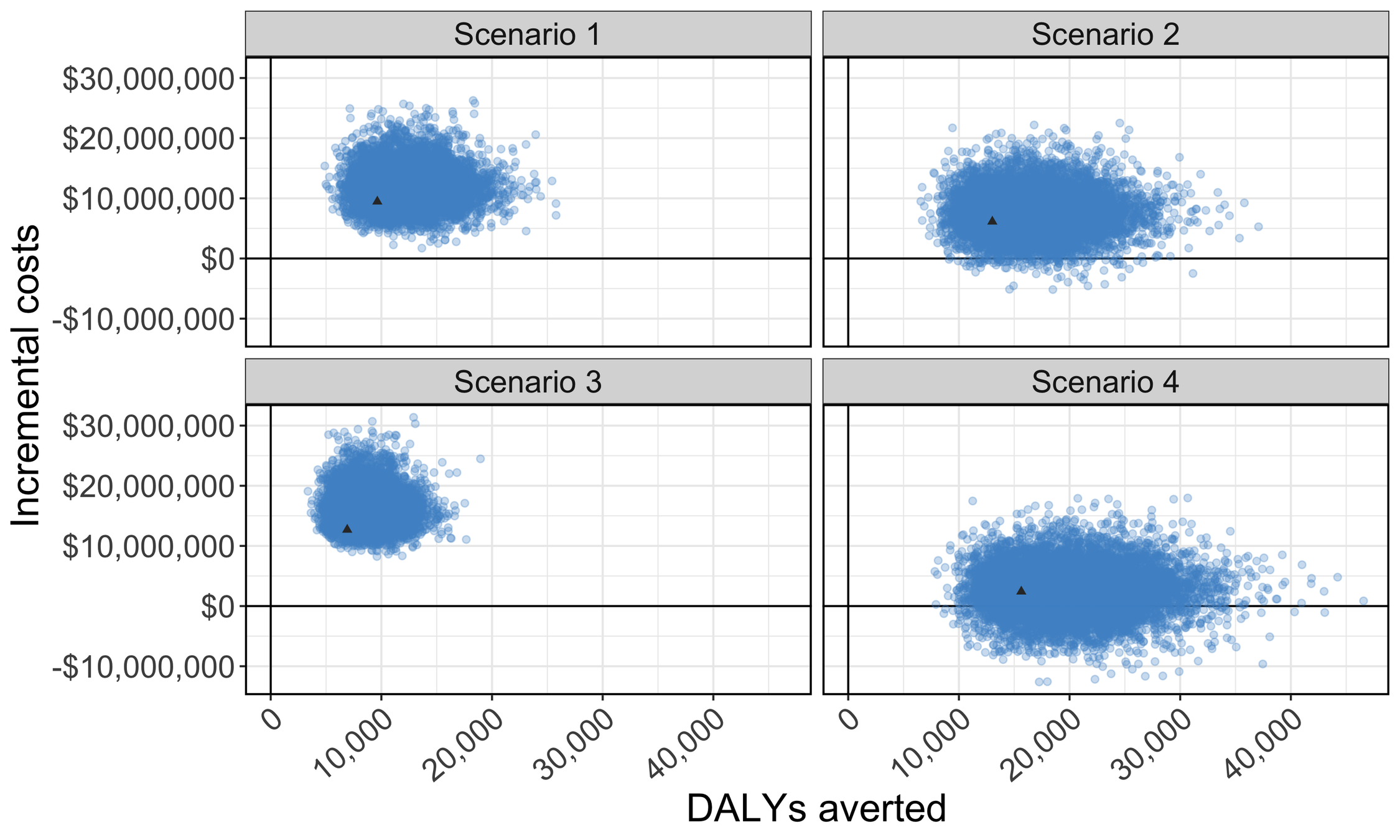

Supplement: S3 Appendix — Results are for Scenario 1 (tafenoquine for adults, primaquine adherence 66.7%), Scenario 2 (tafenoquine for all, primaquine adherence 66.7%), Scenario 3 (tafenoquine for adults, high primaquine adherence of 90%), and Scenario 4 (tafenoquine for adults, low primaquine adherence of 30%) compared to baseline (7-day low-dose primaquine (0.5 mg/kg), adherence set at comparison scenario) overall for Brazil. The base case analysis results are designated by a black triangle in each panel. (TIF) [file pmed.1004255.s004.tif]

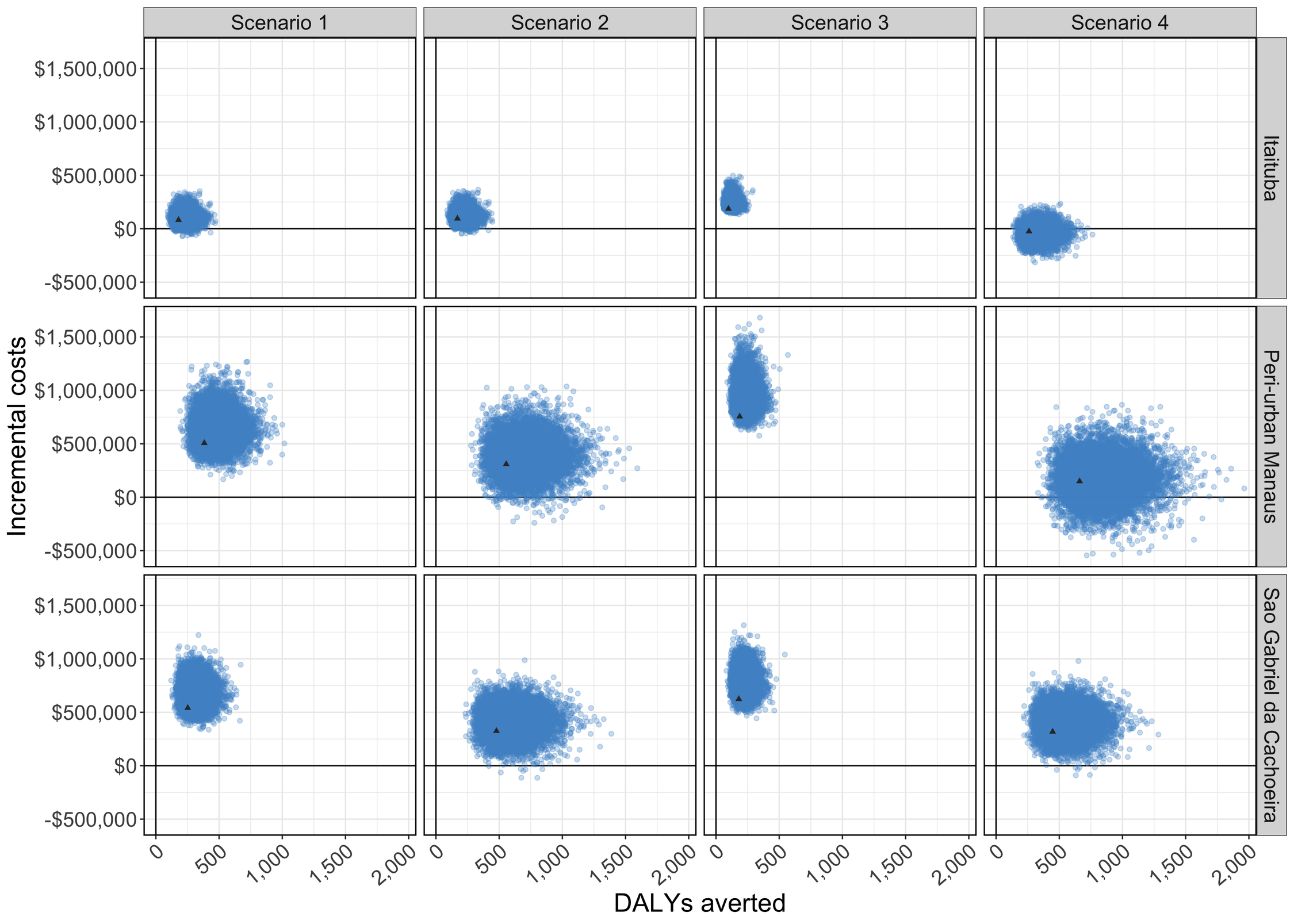

Supplement: S5 Appendix — All scenarios are compared to the baseline scenario for 3 municipalities. The base case analysis results are designated by a black triangle in each panel. (TIF) [file pmed.1004255.s006.tif]

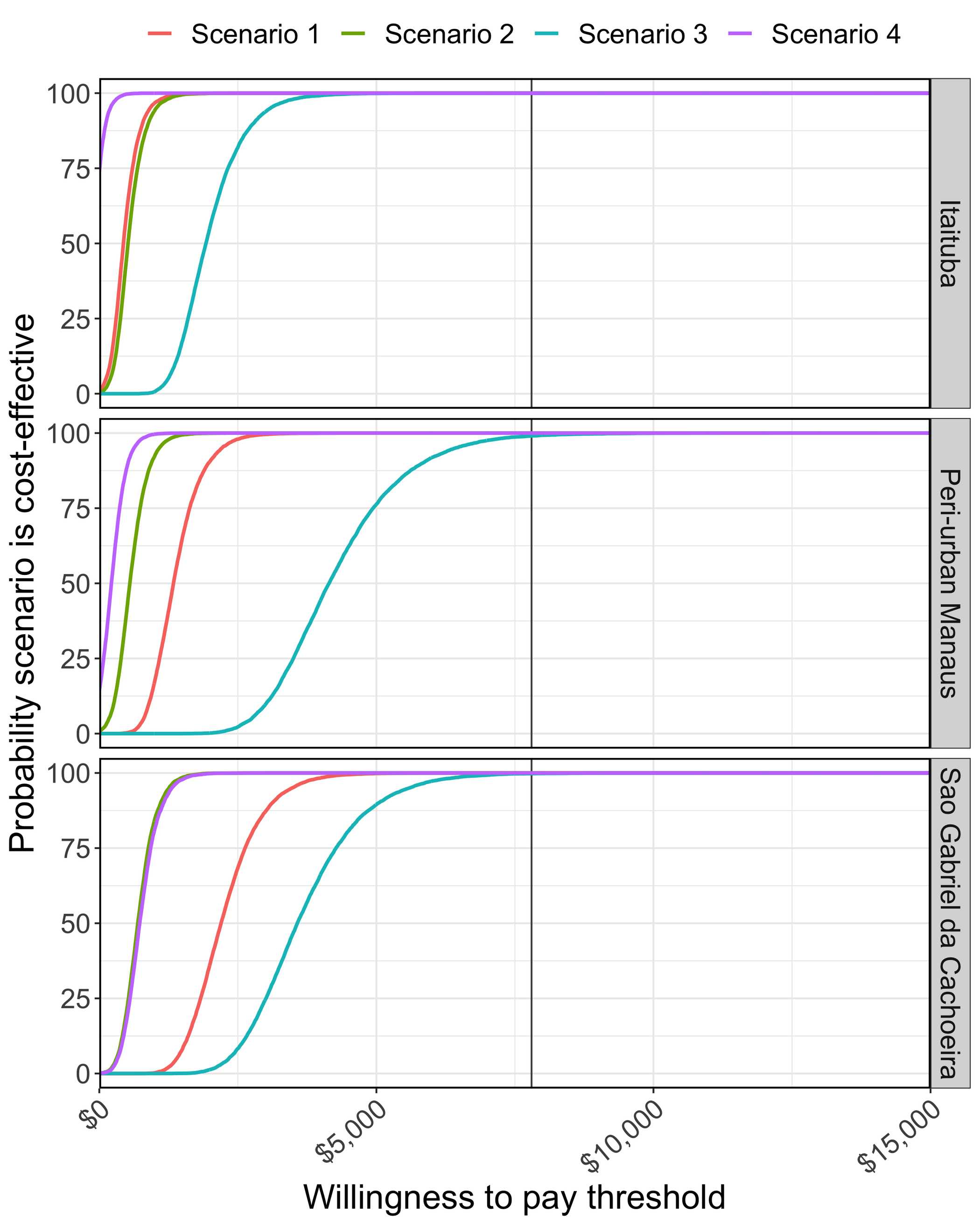

Supplement: S6 Appendix — The black vertical line represents the willingness-to-pay threshold (USD$7,800). (TIF) [file pmed.1004255.s007.tif]
