# Supplementary material for: Tafenoquine following G6PD screening versus primaquine for the treatment of vivax malaria in Brazil: A cost-effectiveness analysis using a transmission model
Source: PLoS Med. 2024 Jan 9;21(1):e1004255. doi: 10.1371/journal.pmed.1004255 (PMC10775976; doi:10.1371/journal.pmed.1004255)
Supplement: S4 Appendix — (DOCX) [file pmed.1004255.s005.docx]

**S4 Appendix.** Mean and 95% credible intervals from the probabilistic sensitivity analysis for disability-adjusted life-years (DALYs) averted, incremental costs, and incremental cost effectiveness ratios (ICERs).

| Scenario | Incremental Costs | DALYs averted | ICER |
| --- | --- | --- | --- |
| 1 | 11,863,099 (6,578,704, 18,409,257) | 12,338 (7,639, 18,245) | 1011 (480, 1,837) |
| 2 | 7,619,267 (1,142,585, 14,633,037) | 16,669 (10,127, 25,014) | 483 (69, 1,052) |
| 3 | 16,173,313 (11,640,655, 22,377,458) | 8,682 (5,469, 12,802) | 1954 (1104, 3,272) |
| 4 | 2,765,773 (-4,867,346, 10,270,936) | 20,120 (12,049, 30,663) | 146 (-255, 589) |
