# Supplementary material for: Tafenoquine following G6PD screening versus primaquine for the treatment of vivax malaria in Brazil: A cost-effectiveness analysis using a transmission model
Source: PLoS Med. 2024 Jan 9;21(1):e1004255. doi: 10.1371/journal.pmed.1004255 (PMC10775976; doi:10.1371/journal.pmed.1004255)
Supplement: Attachment — Submitted filename: price.pdf [file pmed.1004255.s008.pdf]

This paper models the cost effectiveness of a strategy to treat malaria in Brazil under various scenarios.

I was asked for a statistical report and I interpret that to include all aspects of the design and conduct of the study.

Points of detail

Page 6, Table 1 Later on (page 8) we learn that in the sensitivity analysis gamma distributions were used for costs but in the table we have values described as low and high. For transparency and reproducibility I was expecting to find the parameters of the gamma distribution somewhere but I cannot. I assume the values in Table 1 are minima and maxima but I find that hard to square with the use of a gamma distribution which has its support on the non-negative real line. I suspect I have failed to understand something here. Perhaps the authors can clarify?

Page 6 What exactly is an intermediate? This is not a term familiar to me in the diagnostic test literature.

Page 7 What is fadeout here?

Page 7 I know this is picky but if you are going to use the standard BRL for *reais* then why not USD for dollars?

Page 8 Perhaps as a result of my lack of knowledge of malaria epidemiology the explanation of the choice of the three settings needs some more detail.

Page 18 Good to have a list of abbreviations, I wish more people did this, but I notice CEAC seems to be missing (and not defined in the text).

Page 19 Good to provide the code but this is for the transmission model not the economic models as far as I can see. I only checked the R files, I do not read C++.

Summary

Mostly for clarification. I think for reproducibility we need the code for the simulations presented here. I leave it to the clinical reviewers to comment on the plausibility of the chosen parameter values

Michael Dewey
