# Supplementary material for: Tafenoquine following G6PD screening versus primaquine for the treatment of vivax malaria in Brazil: A cost-effectiveness analysis using a transmission model
Source: PLoS Med. 2024 Jan 9;21(1):e1004255. doi: 10.1371/journal.pmed.1004255 (PMC10775976; doi:10.1371/journal.pmed.1004255)
Supplement: Attachment — Submitted filename: R2_review_responses.docx [file pmed.1004255.s009.docx]

**Requests from the editors:**

GENERAL:

Your manuscript has been assessed by four reviewers whose reports can be found below. As you will see from the comments, the reviewers have raised a number of concerns that need addressing. Please carefully revise the manuscript to address all comments raised.

**Author Response**: The comments are all very helpful. Thank you for giving us the opportunity to revise our manuscript.

For in-text reference, citations are placed within square parentheses and should precede punctuation as follows, e.g see line 29 “…. 17.6 million United States Dollars (US$) in 2017 [3].’ Please check and amend throughout.

**Author Response**: This has been done.

Please provide 95% CIs and p values for all results were appropriate, check and amend throughout. For p values, please report these as p<0.001 and where higher as p=0.002 or p=0.050. Suggest reporting statistical information for clarity in the following format: ‘x’; (95% CI [‘y’,’ z’] p<0.001).

**Author Response**: The results presented are for a model-based economic evaluation. P-values are not relevant as there is no hypothesis testing. Where there is uncertainty in estimates in the probabilistic sensitivity analyses, we have presented credibly intervals (CrI).

STUDY DESIGN:

i) For the economic evaluation part of your manuscript, please report your economic analysis according to the appropriate study design provided at <https://www.equator-network.org/?post_type=eq_guidelines&eq_guidelines_study_design=economic-evaluations&eq_guidelines_clinical_specialty=0&eq_guidelines_report_section=0&s=>

and provide the relevant completed checklist. In the checklist please include sufficient text excerpted from the manuscript to explain how you accomplished all applicable items.

**Author Response**: The CHEERS checklist has been included as S1 CHEERS checklist.

ii) For the transmission model, we ask for inclusion of specific items, derived from Geoffrey P Garnett, Simon Cousens, Timothy B Hallett, Richard Steketee, Neff Walker. Mathematical models in the evaluation of health programmes. (2011) Lancet DOI:10.1016/S0140-6736(10)61505-X.

Please ensure all the items listed below are included with your manuscript. Please review the list below and confirm/revise as necessary:

(i) Please provide a diagram that shows the model structure, including how the disease natural history is represented, the process and determinants of disease acquisition, and how the putative intervention could affect the system.

(ii) Please provide a complete list of model parameters, including clear and precise descriptions of each parameter, together with the values or ranges for each, with justification or the primary source cited, and important caveats about the use of these values noted.

(iii) Please provide a clear statement about how the model was fitted to the data.

(iv) For uncertainty analyses, please state the sources of uncertainties quantified and not quantified.

(v) Please provide sensitivity analyses to identify which parameter values are most important in the model. Uncertainty estimates seek to derive a range of credible results on the basis of an exploration of the range of reasonable parameter values. The choice of method should be presented and justified.

(vi) Please discuss the scientific rationale for this choice of model structure and identify points where this choice could influence conclusions drawn. Please also describe the strength of the scientific basis underlying the key model assumptions.

**Author Response**: The focus of this manuscript is the cost effectiveness analysis, rather than transmission model, the development and fitting of which was the focus of two previous manuscripts, including one in PLoS Medicine (Nekkab et al. 2021). However, we recognise the need to include sufficient detail to interpret the results of our cost effectiveness analyses, and so we have expanded our description of the transmission model on lines 98-120 in the Methods section to include further details:

*“Full details of the transmission model can be found in Nekkab et al. [19] and White et al. [20]. Briefly, the individual-based transmission model originally calibrated to epidemiological data from Papua New Guinea (PNG) represents P. vivax transmission, and includes sources of heterogeneity such as individual-level treatment, seasonality in exposure, and age-specific immunity. The model presented in White et al. was extended in Nekkab et al. to the Brazilian context, to capture the two predominant modes of transmission: peri-domestic transmission within and near households, and; occupational exposure predominantly affecting working age males. The model was extended to capture these modes of transmission with: the stratification of individuals in the model as male or female; males further stratified according to their exposure source (peri-domestic or occupational), and; two populations of mosquitoes representing peri-domestic or occupational exposure. The model was calibrated to the Brazilian context using several data sources from Brazil and validated by the National Malaria Control Programme and malaria experts in Brazil. The model was calibrated to case notification data for 126 municipalities for which there were at least 100 cases in 2018, such that heterogeneity in demographics and transmission intensity was represented. The intervention simulations were generated for a population size of 100,000 and subsequently converted to the appropriate population size for each municipality estimated for 2018. The results cover a ten-year time horizon from 2020 to 2029, and assume that G6PD screening is fully rolled out in January 2021.*

*The cost-effectiveness analysis presented herein uses the median value of multiple disease and treatment model states across 100 simulations of the individual-based model over time for each municipality. Specifically, the number of primaquine and tafenoquine doses, cases in males and females over 16 years, cases in males and females under 16 years, number of cases in pregnant women, and G6PD tests were provided by the transmission model. The model assumed a truncated exponential age distribution with a mean age of 32 years.*”

ABSTRACT:

For the abstract, please structure the abstracts along the following 3 headings: Background, Methods and Findings, Conclusions. (Currently there are separate headings for Methods and Findings sections). Please remove all other subheaders.

**Author Response**: This has been amended as requested.

Abstract Background: Provide the context of why the study is important. The final sentence should clearly state the study question.

**Author Response**: This has been edited to: “Malaria transmission modelling has demonstrated the potential impact of semi-quantitative glucose-6-phosphate dehydrogenase (G6PD) testing and treatment with single-dose tafenoquine for Plasmodium vivax radical cure but has not investigated the associated costs. This study evaluated the cost-effectiveness of P. vivax treatment with tafenoquine after G6PD testing using a transmission model.”

Abstract Methods and Findings:

Please quantify the main results with 95% CIs and p values.

**Author Response**: Only the base case ICERs are reported; accordingly, CIs and p-values are not appropriate here. We have clarified that these are the base case results to clarify this point.

In the last sentence of the Abstract Methods and Findings section, please describe the main limitation(s) of the study's methodology.

**Author Response**: This has been added: “The main limitations of this study are due to parameter uncertainty around our cost estimates for low transmission settings, the costs of G6PD screening, and the severity of vivax malaria.”

Abstract Conclusions:

Please interpret the study based on the results presented in the abstract, emphasizing what is new without overstating your conclusions.

**Author Response**: We have adjusted our conclusion to the following: “In our modelling study that incorporated impact on transmission, tafenoquine prescribed after a semi-quantitative G6PD testing was highly likely to be cost-effective in Brazil. These results demonstrate the potential health and economic importance of ensuring safe and effective radical cure.”

AUTHORS SUMMARY:

At this stage, we ask that you include a short, non-technical Author Summary of your research to make findings accessible to a wide audience that includes both scientists and non-scientists. The Author Summary should immediately follow the Abstract in your revised manuscript. This text is subject to editorial change and should be distinct from the scientific abstract. Please see our author guidelines for more information: <https://journals.plos.org/plosmedicine/s/revising-your-manuscript#loc-author-summary>

Ideally each sub-heading should contain 2-3 single sentence, concise bullet points containing the most salient points from your study.

In the final bullet point of ‘What Do These Findings Mean?’ Please include the main limitations of the study in non-technical language.

**Author Response**: This section has been added.

ACKNOWLEDGMENTS/ DECLARATIONS

Please remove all statements apart from acknowledgements, author contributions and abbreviations from the end of the main manuscript and include these only in the relevant parts of the manuscript submission form. Funding, competing interest, and data availability will be compiled as metadata. The ethics declaration should be included in your method section.

**Author Response**: This has been done.

**Reviewer #1**

This is a well-written cost-effectiveness study that makes a single point in an important country for relapsing malaria.  Tafenoquine is a better choice (more cost effective) than reasonable assumptions for the current primaquine regimen.  As the authors mention in the discussion, the analysis shifts when higher dose primaquine regimens are used, but so do the risks.  This study should encourage the Brazilian government to continue its phased roll out of tafenoquine to eliminate vivax malaria in hopes that further practical information will be gained from actual field data for use in other countries / situations.  PLOS states that it also gets statistical reviews for its papers, which is good as although the numbers seem reasonable to me, they need to be examined by an outside statistician.
Author Response: We thank the reviewer for their positive review of our article.

**Reviewer #2**

This is an innovative and interesting article which uses mixed methods to provide robust input to a proven model. It provides guidance as to the use of new technology that seems appropriate for policy action. I think it would help the reading public to have a bit more explanation of two areas that are mentioned but to my mind could be more complete. First a more complete review of the semi-quantitative device utilized including country of origin and cost as well as explicit use parameters i.e. training needed and if there are other requirements to make it operational. Introducing new technology is always a tricky process and the application of implementation science principles might be suggested.
**Author Response**: We have added details on the specific test and associated operational research in the second paragraph of the introduction “The STANDARD G6PD Test (SD Biosensor, Republic of Korea) has demonstrated good diagnostic accuracy [17] and operational feasibility [18].” We’ve also added further details on the training costs in Table 1: “Includes room rental, staff time, stationery, and catering. Assumed two healthcare workers per healthcare facility.”

A second observation that could greatly impact implementation is the mentioned compliance with treatment once cases have been identified. This may require more costly behavioral change programs which do not seem to be reflected in the model. Perhaps a suggestion as to how roll out might be effected given model results and use would be useful to policy makers.
In all a good and interesting article worthy of publication.

**Author Response**: In addition to the previously referred to training, behavioural change for healthcare workers is being implemented through monthly malaria training meetings. These meetings would be implemented regardless of scenario; accordingly, costs would not differ.

**Reviewer #3**

The authors have produced a clear and well written paper to assess the cost effectiveness of rolling out Tafenoquine following G6PD screening vs primaquine for the treatment of vivax malaria in Brazil.
There are three minor comments:
1. While the dynamic transmission model paper is published elsewhere, because the CEA is based on it, it is necessary for the reader to understand the assumptions made for future interventions in the baseline scenario. The transmission model baseline projections should be described in the methods section. This will provide the reader with context for understanding geographical differences in CEA results.

**Author Response**: We appreciate the need for additional detail to interpret the results as presented in this manuscript. In response to the editor’s comments above, we have added further description of the transmission model implementation in the Methods on lines 98-120.

Given the addition of these details, we have also included additional subheadings in the methods to make clearer where the transmission model details are presented, and where the treatment scenarios (including the baseline) are described in text (Treatment Scenarios subsection begins line 124).

2. No information was given on treatment access and how it may differ geographically. If the scenarios assume passive treatment only, then differences in treatment access will impact the analysis.

**Author Response**: While access may differ geographically, this would result in differences across the scenarios since all scenarios assumed passive treatment only. We assumed 95% access to treatment of clinical cases across all municipalities. This was based on expert opinion (Prof Marcus Lacerda). No comprehensive study has yet been conducted to compare access to treatment in different settings in Brazil although expert opinion suggest that treatment access is very high in Brazil since it is free of charge and many individuals are familiar with malaria related symptoms.

3. A section should be added to the discussion on the key sources of variation in the dynamic transmission model and how it impacts the CEA sensitivity analysis and results. It was unclear whether the n random draws from the cost parameter distributions were applied to m transmission model baseline simulations to result in n x m iterations. Please elucidate.
**Author Response**: We thank the reviewer for highlighting our oversight in clearly detailing how the output from the transmission model was used in this cost-effectiveness analysis. As described in response to editor’s comments above, Nekkab et al. simulated the individual-based model 100 times for each scenario and municipality. The present analysis uses the median estimates across each time period (year) of the number of primaquine and tafenoquine doses, cases in males and females over 16 years, cases in males and females under 16 years, number of cases in pregnant women, and G6PD tests, for each scenario. These are the values that are subsequently used in the economic model. That is, the heterogeneity across municipality, as represented by varying demographics and transmission intensities is captured, but the variability within each municipality is not considered in this analysis. Subsequently, the sensitivity analyses explored in this work considers n draws from the parameter distributions for the transmission model median output. We have added clarification to line 215 of the Methods, which now reads:

*“The PSA model parameters were each applied to the transmission model output for each municipality for the four scenarios…”*

In the Discussion (lines 358-367) where we described the limitations associated with the absence of variability in low transmission settings, we have added a sentence such that it now reads:

“*For very low transmission settings, stochastic noise and fadeout result in more unstable transmission dynamics and greater variation between simulations. In addition, calibrated incidence per 1000 population for these low transmission settings assumes homogenous mixing in the population and no importations to sustain transmission; therefore, local transmission dynamics in communities are likely to differ compared to the simplified model aggregated municipality-level assumptions. Consequently, projected DALYs for these settings are less reliable and should be interpreted with caution. The absence of this variation in model output has minimal impact on municipalities with higher transmission (and thus at a national level) where the results converge to those from a deterministic model [19], and where the majority of the costs would be accrued.*”

**Reviewer #4**

This paper models the cost effectiveness of a strategy to treat malaria in Brazil under various scenarios. I was asked for a statistical report and I interpret that to include all aspects of the design and conduct of the study.

Points of detail

Page 6, Table 1 Later on (page 8) we learn that in the sensitivity analysis gamma distributions were used for costs but in the table we have values described as low and high. For transparency and reproducibility I was expecting to find the parameters of the gamma distribution somewhere

but I cannot. I assume the values in Table 1 are minima and maxima but I find that hard to square with the use of a gamma distribution which has its support on the non-negative real line. I suspect I have failed to understand something here. Perhaps the authors can clarify?

**Author Response**: The upper and lower values for each parameter are used to fit a specified distribution by finding the parameter values for the distribution that minimise the difference between the lower/upper values and the 2.5- and 97.5-percentiles of that distribution (i.e., representing a 95% interval). We have added these details to Table 1 including the baseline, lower and upper values, the specified distribution, and the parameters for these fitted distributions. We have added additional clarification to the Methods on lines 201-214, which now reads:

*“A one-way sensitivity analysis and probabilistic sensitivity analysis (PSA) was run on all parameters in the cost-effectiveness analyses. Table 1 contains a list of all parameters, with the point estimate used as baseline, and lower and upper values used directly in the one-way sensitivity analysis. For the PSA, the point estimate, lower and upper values were used to fit appropriate distributions from which to sample (i.e., by matching the lower and upper values to 2.5- and 97.5-percentiles, respectively). Gamma distributions were used for costs and DALY weights, and beta distributions were used for all other parameters except for the lifetime of the semi-quantitative machine. For this parameter we assumed a normal distribution truncated below at 1 year, on the assumption that the average semi-quantitative device lifetime was at least 1 year. A range of two to ten years was used for the lifetime of the semi-quantitative machine in the one-way sensitivity analysis. Table 1 also contains the two fitted distributional model parameters for each cost-effectiveness analysis model parameter.”*

Page 6 What exactly is an intermediate? This is not a term familiar to me in the diagnostic test literature.

**Author Response**: We have added further details about intermediate deficiency in the second paragraph of the introduction: “*The threshold for G6PD deficiency for prescribing tafenoquine, however, is set at 70%, which requires semi-quantitative screening in order to exclude individuals with intermediate G6PD deficiency (30-70% activity).*”

Page 7 What is fadeout here?

**Author Response**: Fadeout here is an epidemiological term referring to cases/infections reaching zero. We have added the following brief explanation to lines 195-197, at first mention of this term: *“…, and fadeout (i.e., case counts reaching zero as a result of fluctuations at low case numbers, and in the absence of importation) dominated the low numbers of cases in the transmission model simulations.*”

Page 7 I know this is picky but if you are going to use the standard BRL for reais then why not USD for dollars?

**Author Response**: BRL has been replaced with Brazilian reais where mentioned.

Page 8 Perhaps as a result of my lack of knowledge of malaria epidemiology the explanation of the choice of the three settings needs some more detail.

**Author Response**: We have expanded further on the different transmission settings that these three municipalities represent, as described in Nekkab *et al*.

“*To provide insights into a range of epidemiological transmission settings, the results of the sensitivity analyses are presented for three municipalities: peri-urban Manaus, São Gabriel da Cachoeira, and Itaituba. The first two municipalities are in the state of Amazonas, while Itaituba is in Pará state. Peri-urban Manaus excludes the malaria-free urban areas of that municipality. These municipalities were selected as exemplars of where occupational exposure (Itaituba) or peri-domestic transmission (Sao Gabriel da Cachoeira) were the dominant modes of transmission, or a combination of both modes (peri-urban Manaus). Transmission intensity was 23 cases per 1000 person years in Itaituba, 114/1000 in peri-urban Manaus, and 267/1000 in São Gabriel da Cachoeira [18].*”

Page 18 Good to have a list of abbreviations, I wish more people did this,

but I notice CEAC seems to be missing (and not defined in the text).

**Author Response**: Good spot! We’ve removed this abbreviation from the text since not used frequently.

Page 19 Good to provide the code but this is for the transmission model not the economic models as far as I can see. I only checked the R files, I do not read C++.

**Author Response**: We have provided the code to implement the cost-effectiveness model for the baseline and each tafenoquine treatment scenario in Appendix 1.

Summary

Mostly for clarification. I think for reproducibility we need the code for the simulations presented here. I leave it to the clinical reviewers to comment on the plausibility of the chosen parameter values.
